# Emerging Biomarkers for Predicting Bladder Cancer Lymph Node Metastasis

**DOI:** 10.3389/fonc.2021.648968

**Published:** 2021-03-19

**Authors:** Chunyu Zhang, Jiao Hu, Huihuang Li, Hongzhi Ma, Belaydi Othmane, Wenbiao Ren, Zhenglin Yi, Dongxu Qiu, Zhenyu Ou, Jinbo Chen, Xiongbing Zu

**Affiliations:** ^1^ Department of Urology, Xiangya Hospital, Central South University, Changsha, China; ^2^ Department of Radiation Oncology, Hunan Cancer Hospital, Central South University, Changsha, China; ^3^ George Whipple Lab for Cancer Research, University of Rochester Medical Institute, Rochester, NY, United States

**Keywords:** lymph node metastasis, bladder cancer, biomarkers, oncogenes, tumor suppressor genes

## Abstract

Bladder cancer is one of the leading causes of cancer deaths worldwide. Early detection of lymph node metastasis of bladder cancer is essential to improve patients’ prognosis and overall survival. Current diagnostic methods are limited, so there is an urgent need for new specific biomarkers. Non-coding RNA and m6A have recently been reported to be abnormally expressed in bladder cancer related to lymph node metastasis. In this review, we tried to summarize the latest knowledge about biomarkers, which predict lymph node metastasis in bladder cancer and their mechanisms. In particular, we paid attention to the impact of non-coding RNA on lymphatic metastasis of bladder cancer and its specific molecular mechanisms, as well as some prediction models based on imaging, pathology, and biomolecules, in an effort to find more accurate diagnostic methods for future clinical application.

## Introduction

Bladder cancer (BCa) is the 10th most common cancer form, causing an estimated 549,000 new cases and 200,000 deaths in 2018. The incidence of BCa in men is four times that of women, and smoking is the most important risk factor for BCa in the population (1). More than 90% of bladder cancers are urothelial carcinoma, and the rest are squamous cell carcinoma and adenocarcinoma.

The most common metastatic manner of BCa is lymph node metastasis (LNM), which is more common in pelvic lymph nodes. LNM has a great influence on the prognosis and survival rate of BCa patients. For BCa patients with positive LNM, the 5-year CSS rate was 27.7%, which is significantly lower than that of patients without lymph node metastasis (2). CT or MRI is commonly used in clinical practice to diagnose pelvic LNM, but it is often difficult to accurately detect metastatic lymph nodes less than 6.8 mm in diameter (3). Many studies have recently reported the correlation between molecular markers and BCa metastasis, indicating a direct link between LNM and abnormal expression of specific biomarkers. Therefore, high-risk LNM patients can be diagnosed by detecting specific biomarkers to achieve early detection and early treatment, thereby achieving timely treatment and improving the survival rate.

**Figure 1:**
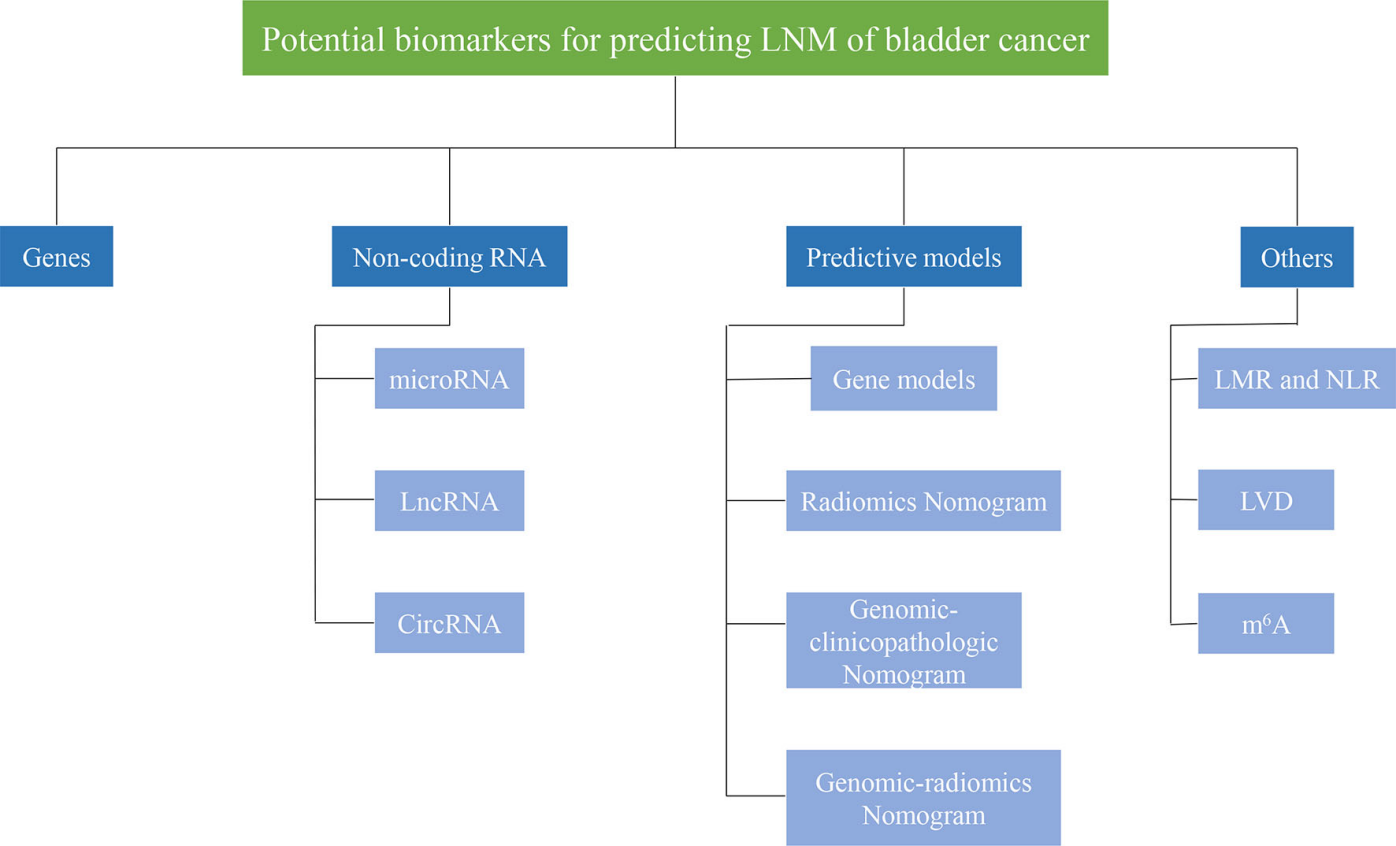
Potential biomarkers for predicting lymph node metastasis of bladder cancer. Biomarkers for predicting lymphatic metastasis of bladder cancer can be divided into different categories, such asgenes, non-coding RNA, prediction models.

Moreover, some predictive models, including imaging, pathology, and molecular markers, have been gradually developed and verified. In this review, we summarized the markers for LNM in BCa from different aspects, including genes, non-coding RNA, and some predictive models (**Figure 1**). The downstream genes of non-coding RNA are specifically listed here (**Table 1**). Generally, mechanisms for LNM in cancers mainly include cell proliferation, cell invasion and migration, inhibition of cell apoptosis, and chemosensitivity. Based on this, we also elaborated on the regulation mechanism of these biomarkers.

**Table 1:** Downstream genes of non-coding RNA in bladder cancer.

Marker	Relationship with downstream genes	Downstream genes	Reference
miR-101	Negative	FZD4	(4)
		c-FOS	(5)
		c-Met	(6)
		VEGF-C	(7)
		COX-2	(8)
miR-143	Negative	COX-2	(9)
		MSI2	(10)
miR-133b	Positive Negative	DUSP1	(11)
		Bcl-wˎAkt1	(12)
		Epidermal growth factor receptor	(13)
		TAGLN2	(14)
miR-539	Negative	IGF-1RˎAKTˎERK	(15)
miR-497	Positive	E-cadherin	(16)
	Negative	Vimentin	
		BIRC5ˎWNT7A	(17)
		E2F3	(18)
miR-154	Negative	RSF1ˎRUNX2	(19)
		ATG7	(20)
miR-223	Positive	Caspase-3/7	(21)
	Negative	WDR62	
		ANLN	(22)
		Nuclear receptor co-activator 1	(23)
miR-148a	Negative	DNMT1	(24)
miR-3658	Positive	LASS2	(25)
LncRNA MALAT1	Negative	E-cadherin	(26)
	Positive	ZEB1, ZEB2	
		VEGF-C	(27)
		Bcl-2ˎMMP-13	(28)
		Foxq1	(29)
		Cyclin D1	(30)
LncRNA PVT1	Positive	VEGF-C	(31)
		CDK1	(32)
LncRNA OXCT1-AS1	Positive	JAK1	(33)
LncRNA BLACAT2	Positive	VEGF-C	(34)
LncRNA LNMAT1	Positive	CCL-2ˎVEGF-C	(35)
LncRNA SNHG16	Positive	ZEB1ˎ ZEB2	(36)
		TIMP3	(37)
		STAT3	(38)
LncRNA ZFAS1	Positive	ZEB1ˎZEB2	(39)
	Negative	KLF2ˎNKD2	
LncRNA DLX6-AS1	Positive	HSP90B1	(40)
		Wnt/β-catenin	(41)
LINC01296	Positive	EMT	(42)
LncRNA DANCR	Positive	CCND1ˎPLAU	(43)
		MSI2	(44)
LncRNA SPRY4-IT1	Positive	EZH2	(45)
LncRNA NNT-AS1	Positive	HMGB1	(46)
		PODXL	(47)
LncRNA LNMAT2	Positive	PROX1	(48)
LncRNA HOXA-AS2	Positive	Smad2	(49)
LncRNA HNF1A-AS1	Positive	Bcl-2	(50)
CircHIPK3	Negative	HPSEˎMMP-9ˎVEGF	(51)
CircFNDC3B	Negative	G3BP2/SRC/FAK	(52)
CircFUT8	Positive	KLF10	(53)
CircACVR2A	Positive	EYA4	(54)
CircPICALM	Positive	STEAP4ˎEMT	(55)
cTFRC	Positive	TFRC	(56)

## The Molecular Function of Genes in BCa With LNM

**Figure 2:**
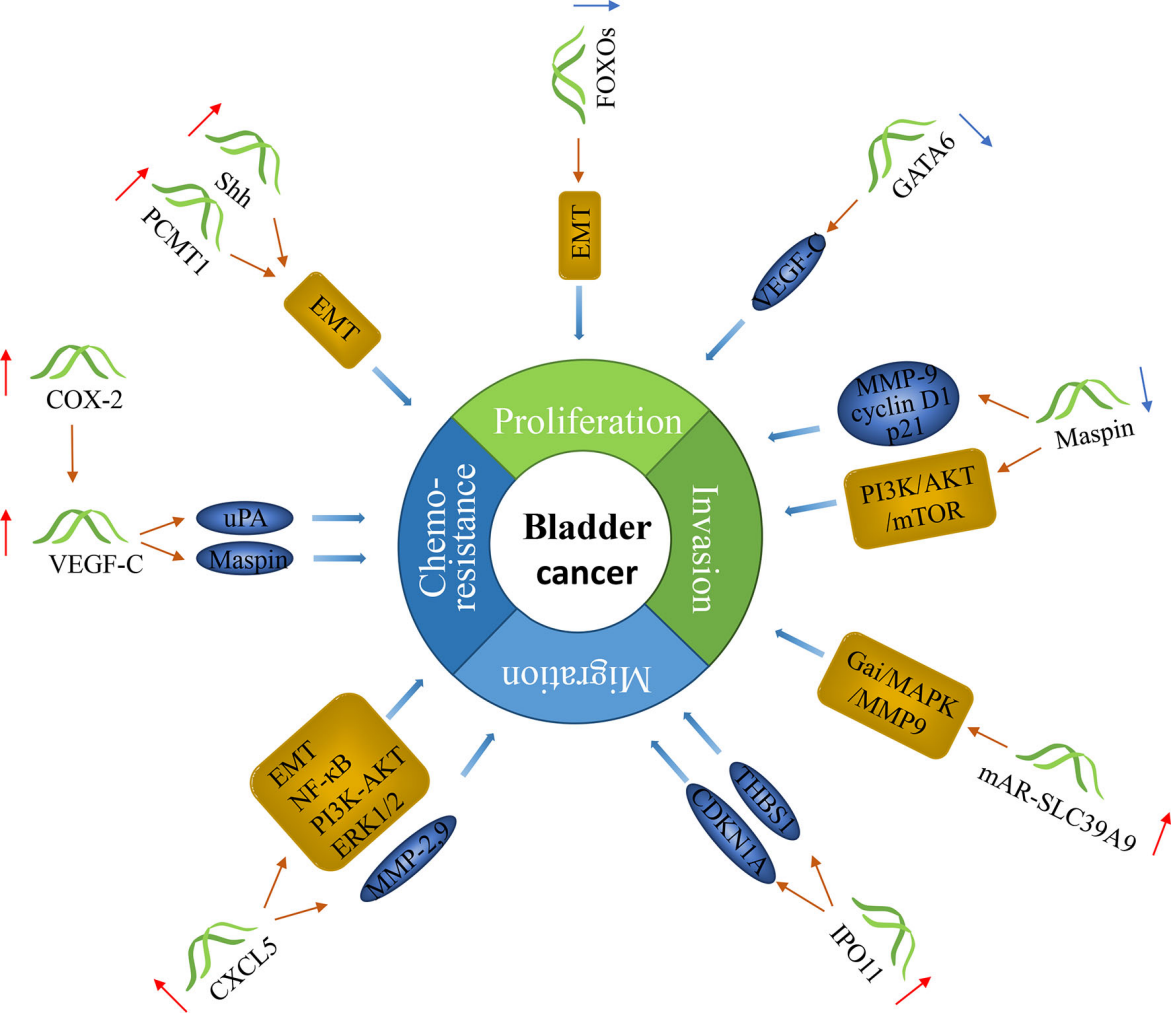
The molecular function of genes in bladder cancer with lymph node metastasis. Genes can predict lymph node metastasis in bladder cancer. Some of them can promote the progression of cancer, and some can inhibit it.

There have been many studies on genes as markers for lymph node metastasis in bladder cancer. These genes act as oncogenes or tumor suppressor genes to influence the progression of cancer (**Figure 2**).

### Genes as Oncogenes

VEGF-C (vascular endothelial growth factor C) is the first discovered lymphangiogenesis factor. It contains the mature form of the VEGF homology region. Our team’s studies found that the expression of VEGF-C in BCa patients with LNM was significantly higher than that in BCa patients without LNM (57). Simultaneously, we also found that VEGF-C can promote proliferation, invasion, metastasis, and mitomycin C resistance of BCa cells. The mechanisms for that are thought to be related to the increased ratio of Bcl-2/Bax, inactivation of Caspase-3, and increased expression of MMP-9. Also, phosphorylated p38 MAPK and Akt, Keratin 8, Serpin B5, and Annexin A8 may be involved (58, 59). VEGF-C can promote the formation of tumor lymphatic vessels and the metastasis of tumor cells to regional lymph nodes. The combination of the activated VEGF-C and VEGFR-3 can induce phosphorylation of tyrosine kinase, causing the proliferation of lymphatic endothelial cells, thereby promoting the proliferation or expansion of lymphatic vessels (60). VEGF-C also positively affected primary tumor cells’ invasiveness since it changed the adhesion of tumor cells to the extracellular matrix, thereby providing the necessary environmental conditions for tumor cells to more easily transfer to the surrounding extracellular matrix. VEGF-C can stimulate lymphatic endothelial cells to release proteolytic enzymes, such as uPA, which facilitate the invasion and infiltration of cancer cells into the matrix, making cancer cells more easily detached from the original tissue (61). The up-regulation of VEGF-C may be the reason for BCa cells’ resistance to cisplatin, and the inhibition of VEGF-C reverses the resistance by increasing the expression level of maspin (62). Therefore, we suggest that VEGF-C and VEGFR-3 expression may serve as new indicators for early detection and diagnosis of BCa lymphatic metastasis in the future. Additionally, COX-2 may stimulate VEGF-C secretion to promote the formation of lymphatic vessels (63). COX-2, a subtype enzyme in the COX family, is an inducible enzyme. COX (Cyclooxygenase) is a rate-limiting enzyme in prostaglandin synthesis, which can catalyze arachidonic acid metabolites to prostaglandins. Previous studies have shown that COX-2 expression was significantly increased in BCa tissues and was associated with LNM (64).

Another well-known gene that functions as an oncogene in BCa is PCMT1. PCMTl gene is located at 6p22.3-6q24, about 60kb in length, and contains eight exons and seven introns. Studies have shown that the expression of PCMT1 in BCa tissue was higher than that in normal urothelial tissue, and its expression was significantly associated with LNM. PCMT1 regulated the migration and invasion of BCa cells by regulating the expression of epithelial-mesenchymal transition (EMT) related genes, such as E-cadherin, vimentin, Snail, and Slug (65). Sonic Hedgehog (Shh) also activated EMT to promote tumorigenicity and stemness in BCa (66). Shh is a member of the Hedgehog (HH) family. The study found that the expression of Shh protein was significantly correlated with LNM (67). Shh can promote the migration and invasion of BCa cells. The Shh pathway’s activation through the binding of the Shh ligand to the transmembrane protein Patched1 eliminates the inhibitory effect on smoothened (SMO). The activation of SMO produced a downstream signaling cascade that led to the nuclear translocation of the transcription factor Gli1, which further induce the transcription of target genes (68).

The overexpression of CXCL5 can promote the progression of BCa. CXCL5, known as epithelial-derived neutrophil-activating peptide 78 (ENA78), is a small (8-14 kDa) protein belonging to the CXC-type chemokine family. CXCL5 (chemokine C-X-C motif ligand 5) was expressed higher in BCa tissues than normal tissues, which was associated with LNM (69). It is also related to promoting mitomycin resistance by activating EMT and NF-κB pathway (70). Moreover, CXCL5 increased BCa cells proliferation, migration, and decreased cell apoptosis through Snail, PI3K-AKT, and ERK1/2 signaling pathways. In addition, CXCL5 combined with CXCR2 induces the expression of MMP-2 and MMP-9 and activates the PI3K/AKT signaling pathway (71, 72). Matrix metalloproteinases (MMPs) are a family of structurally related zinc-dependent endopeptidases that can substantially degrade all components of the extracellular matrix (ECM). MMP2, MMP7, and MMP9 are important members of the matrix metalloproteinase family. MMP-2 can physiologically degrade type IV collagen. Mohammad et al. (73) found that the higher the MMP-2 activity level in BCa, the higher the positive rate of LNM. MMP-7, also known as matrilysin, is the smallest MMP. It is produced by the tumor cells themselves, unlike other MMPs which are solely produced by stromal cells. Studies have shown that high expression of MMP-7 was significantly associated with LNM of BCa (74). Studies have shown that MMP-9 genes and proteins’ expression levels in urine and blood of patients with BCa were significantly increased (75). These genes can also decompose the extracellular matrix, make cancer cells easily pass through the extracellular matrix, and promote tumor metastasis.

In addition, Zhao et al. (76) identified a new oncogene candidate, IPO11, in BCa, which is located on chromosome 5q12. Importin-11, a 116 kD protein, is encoded by IPO11. It is a karyopherin family member, which mediates the nucleocytoplasmic transport of proteins and nucleic acids through the nuclear pore complexes. Studies have shown that IPO11 mRNA was highly expressed in invasive BCa cell lines. The overexpression of importin-11 was positively correlated with LNM. Importin-11 can promote BCa cells’ invasiveness, which may be related to the abnormal expression of CDKN1A and THBS1 (77). Presler et al. (78) found that SCD1 was overexpressed in BCa, which was related to LNM. SCD-1 (Stearoyl-CoA desaturase-1) can convert SFA (saturated fatty acids) to MUFA (monounsaturated fatty acids). It is located on chromosome 10q24.31. SCD inhibitors and SCD gene interference reduced the proliferation and invasion of BCa cells (79). FGFR3 (fibroblast growth factor receptor 3) stimulated SCD1 activity to promote tumor growth in BCa cells (80).

The studies of our team also found some new oncogenes. ISYNA1 (Inositol-3-phosphate synthase 1) was positively associated with tumor T stage and LNM of BCa patients. It is an important regulatory factor in promoting proliferation and inhibiting apoptosis in BCa cells (81). The high expression of mAR-SLC39A9 was directly associated with BCa pathological stage, pathological grade, and lymph node metastasis presence. It also increased BCa metastasis through Gαi/MAPK/MMP9 signaling (82).

### Genes as Tumor Suppressors

Maspin (mammary sefine protease inhibitor) is an important member of the serin protease inhibitor (serpin) superfamily. It is located at 18q21.3-q23. Our team’s previous studies found that Maspin expression in BCa tissue was significantly down-regulated in comparison with normal tissues adjacent to the cancer and was related also to LNM. The negative correlation between the protein expression level and VEGF-C is statistically significant (83, 84). Maspin can inhibit the invasion of BCa cells, and its growth-inhibiting properties were related to its localization in cells. The surface-bound Maspin directly controlled the adhesion of BCa cells to the blood vessel wall (85). The combination of nuclear-localized maspin and chromatin can effectively prevent cell migration. Mapsin mainly promoted the development of BCa through DNA methylation and histone deacetylation to cause low expression of genes (86). Maspin modulated HDAC1 target genes, including cyclin D1, p21, MMP9, and vimentin (87). In our previous study, maspin could enhance Cisplatin chemosensitivity through the PI3K/AKT/mTOR signaling pathway in MIBC T24 and 5637 cell lines (88).

Another gene that functions as a tumor suppressor in BCa is GATA6. GATA6 (GATA-binding factor 6), a zinc-finger transcription factor, is located at 18q11.2. It regulates transcription cofactors and RNA polymerase II to the proximal promoter to regulate target genes’ transcription. Wang et al. (89) found that GATA6 decreased in BCa, and further decreased in patients with positive LNM. GATA6 was significantly down-regulated in BCa through frequent promoter methylation. GATA6 mainly inhibited LNM of BCa by regulating VEGF-C. Down-regulation of GATA6 promoted VEGF-C transcription, which promoted lymphangiogenesis, resulting in an increased lymphatic spread of BCa. This increased spread shows that it is of great significance to check the methylation status of the GATA6 promoter in the urine of BCa patients. The low expression of FOXOs was also associated with LNM in BCa (90). FOXO (Forkhead box class O) is the subgroup O of forkhead box (FOX) transcription factors, which has four members, FOXO1, FOXO3, FOXO4 and FOXO6. FOXOs have a highly conserved forkhead DNA binding domain. FOXOs can inhibit the invasion of BCa cells by down-regulating Twist2 and YB-1 and up-regulating E-cadherin (91).

## Regulation of microRNAs for BCa Patients With Lymph Node Metastasis

**Figure 3:**
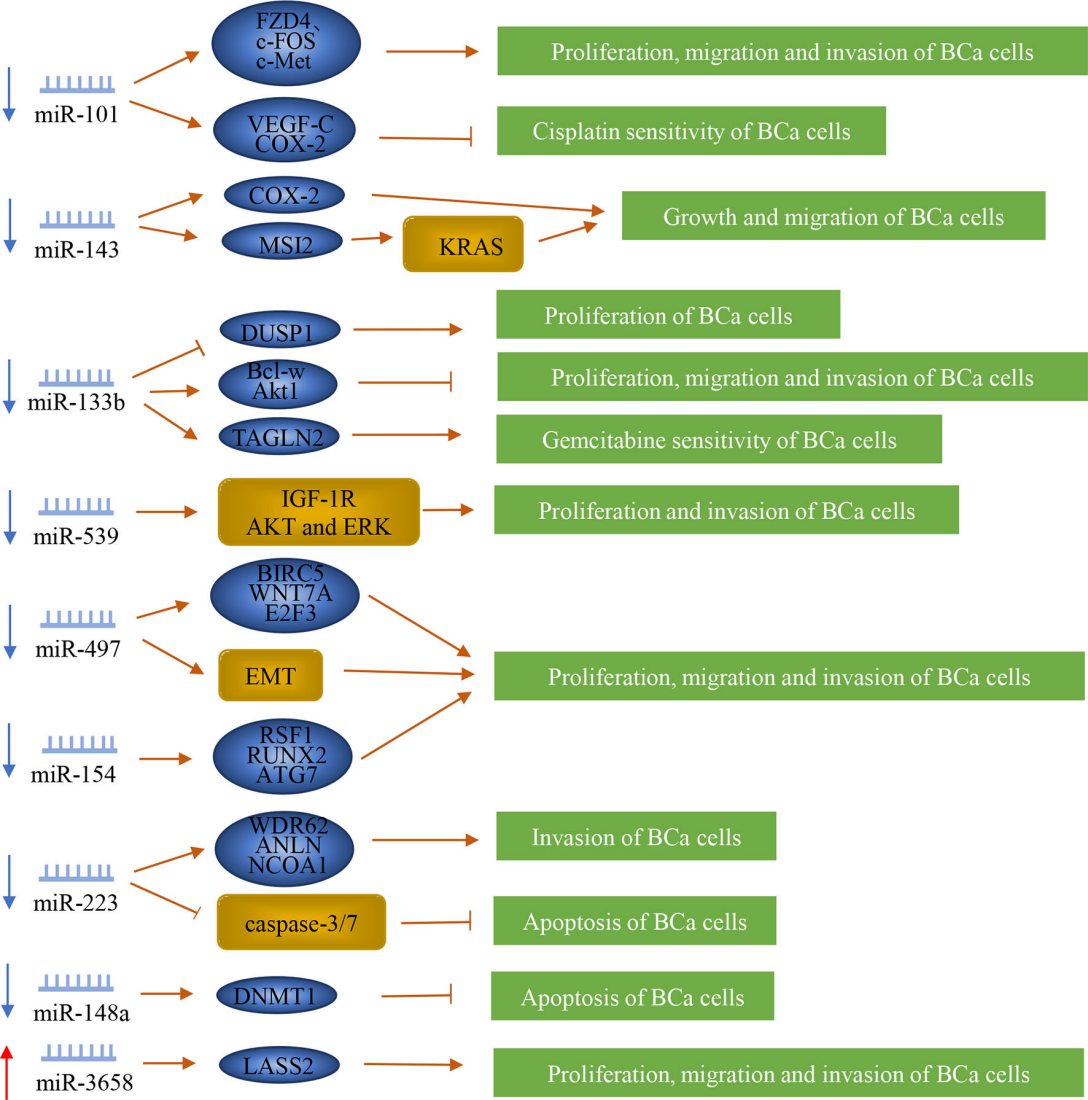
Regulation of microRNAs in bladder cancer patients with lymph node metastasis. MiRNAs play a vital role in the lymph node metastasis of bladder cancer. They can promote or inhibit the metastasis of bladder cancer by regulating downstream genes or proteins.

MiRNA is a type of 21-23nt small RNA, which can complement mRNA and either silence it or degrade it. Most miRNAs are down-regulated in bladder cancer. Moreover, they inhibit the lymph node metastasis of bladder cancer (**Figure 3**).

MiR-101 can suppress the progression of BCa. Studies have shown that the expression of miR-101 in BCa patients was down-regulated and significantly associated with LNM (92). Moreover, it can inhibit the proliferation, migration, and invasion of BCa cells by directly targeting FZD4 (frizzled class receptor 4), c-FOS, and c-Met (4–6). MiR-101 increased Cisplatin sensitivity by inhibiting the expression of VEGF-C and COX-2 in BCa cells (7, 8). MiR-143 also inhibited the growth and migration of BCa cells by targeting COX-2 (9). MiR-143 was reported to suppress the progression of BCa as well and it is located on chromosome 5q32. Liu et al. (93) found that miR-143 was down-expressed in the serum of BCa patients with LNM. It also directly affected the expression of MSI2 through its RNAi effect, which also effectively inhibited the KRAS network, thereby regulating BCa cells (10).

Another gene, miR-133b, is located on chromosome 6p12.2. Studies have shown that the expression level of miR-133b in BCa tissues is significantly reduced, which was significantly correlated with LNM (94). MiR-133b may inhibit the proliferation of BCa by up-regulating dual-specificity protein phosphatase 1 (DUSP1) (11). It inhibited angiogenesis and enhanced BCa cells’ chemosensitivity to Gemcitabine by targeting transgelin 2 (TAGLN2) (14). MiR-133b can regulate the proliferation, migration, and invasion of BCa cells by down-regulating Bcl-w, Akt1, and epidermal growth factor receptor along with its downstream effector protein (12, 13). Liao et al. (15) found that miR-539 was down-regulated in BCa, and was related to LNM. MiR-539 is located on chromosome 14q32.31, and it can inhibit the proliferation and invasion of BCa cells by directly targeting IGF-1R and inactivating the AKT and ERK signaling pathways.

MiR-497 is also known as a tumor suppressor in BCa, and it is located on chromosome 17p13.1. Studies have revealed that the expression of miR-497 in BCa tissue was lower than that of adjacent non-cancer tissues, and it was correlated with LNM (16). MiR-497 can inhibit the proliferation, migration, and invasion of BCa by up-regulating E-cadherin and down-regulating vimentin, α-smooth muscle actin, BIRC5, WNT7A, and E2F3 (16–18). Previous studies have found that miR-154 was significantly down-regulated in BCa tissues and was associated with LNM. MiR-154 is located in the human imprinted 14q32 domain. MiR-154 inhibited the proliferation, migration, and invasion of BCa cells by regulating the expression of RSF1, RUNX2, and ATG7 (19, 20). MiR-223 is located on chromosome Xq12. Sugita et al. (21) found that the expression level of miR-223 was significantly reduced in BCa tissues, which was related to LNM. MiR-223 inhibited cell invasion and promoted cell apoptosis in BCa *via* caspase-3/7 activation and negatively regulating WDR62 (WD repeat domain 62), ANLN, and nuclear receptor coactivator 1 (21–23). MiR-148a, with 68 nucleotide sequences, locates to 7p15.2, and is confirmed by Ma et al. (95) that its expression level in BCa tissue is lower than that of adjacent normal tissues, and that its low expression level is associated with advanced tumor progression and LNM. Also, Lombard et al. (24) found that miR-148a increased the apoptosis of BCa cells by reducing the expression of DNA methyltransferase 1 (DNMT1).

MiR-3658 is known as an oncogene in BCa. The expression of miR-3658 in BCa tissue was up-regulated, and its expression was significantly related to the lymph node infiltration, distant metastasis, and TNM stage (96). It can also promote cell proliferation, migration, and invasion by targeting LASS2 (25).

## LncRNAs Regulate Lymph Node Metastasis in BCa

**Figure 4:**
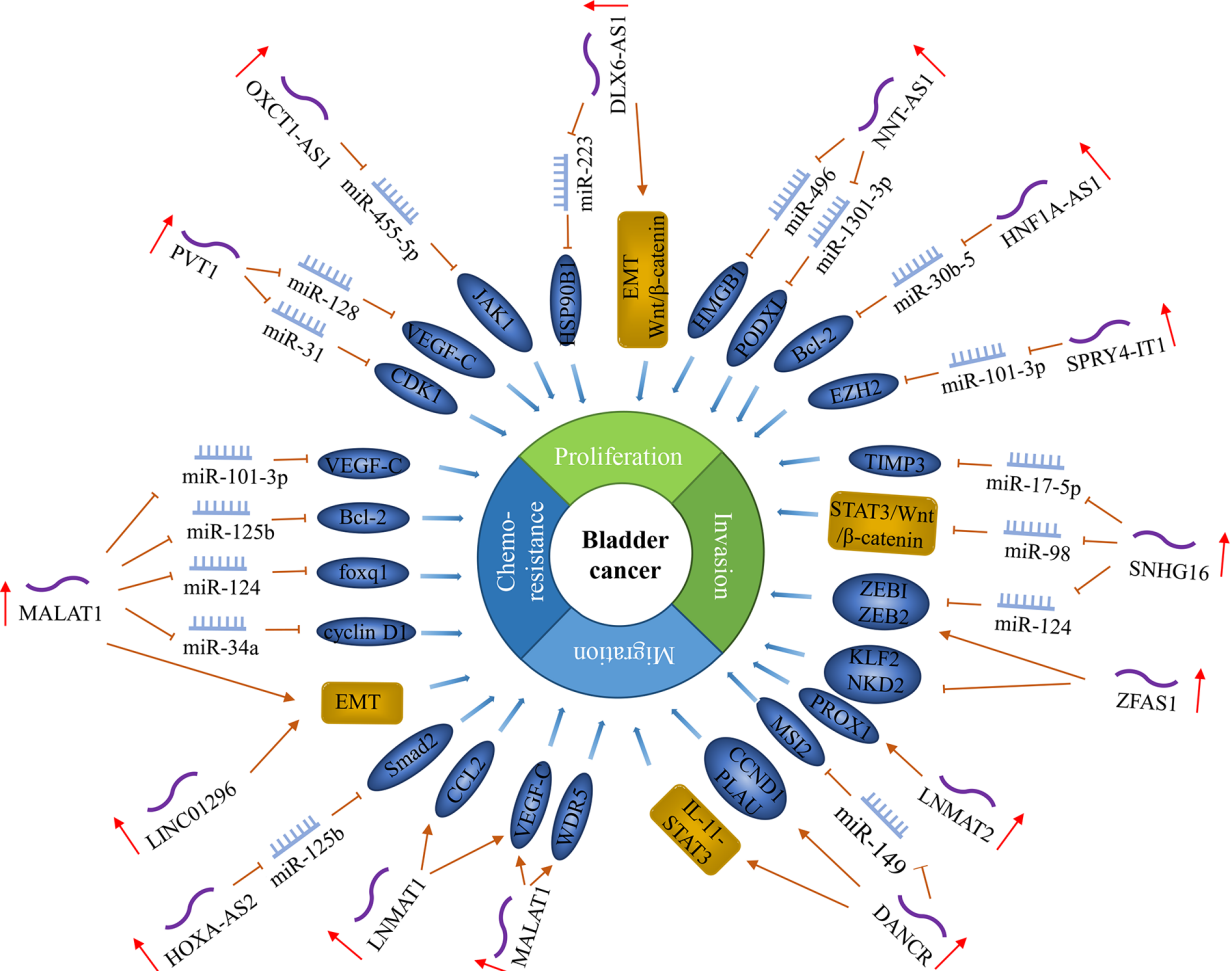
LncRNAs regulate lymph node metastasis in bladder cancer. In bladder cancer, the expression level of some lncRNAs is related to lymph node metastasis and regulates lymph node metastasis by regulating cancer cell proliferation, metastasis, invasion, and chemosensitivity.

LncRNA is a non-coding RNA with a length of more than 200 nucleotides and is closely related to cancer occurrence and development. It can directly bind to proteins to block its functions or change its cellular location, regulate mRNA translation and act as a miRNA sponge. Most lncRNAs act as oncogenes to promote lymphatic metastasis of bladder cancer (**Figure 4**).

Our team’s studies found several lncRNAs as oncogenes, such as MALAT1, PVT1, and OXCT1-AS1. The expression of MALAT1 was positively associated with LNM in BCa. It enhanced the Cisplatin resistance of the BCa cells by regulating the miR-101-3p/VEGF-C pathway (27, 97). MALAT1 promoted proliferation and invasion by miR-125b-Bcl-2/MMP-13, miR-124/foxq1 and microRNA-34a/cyclin D1 in BCa cells (28–30). It also up-regulated EMT-associated ZEB1, ZEB2, and Slug and downregulated E-cadherin levels (26). LncRNA PVT1 is located at 8q24, downstream of MYC. High PVT1 expression is associated with higher tumor stage and positive lymph node metastasis (98). PVT1 directly interacted with miR-128, reducing the binding of miR-128 to VEGF-C, thereby inhibiting the degradation of VEGFC mRNA by miR-128 (31). Moreover, PVT1 down-regulated miR-31 to enhance CDK1 expression and promote the proliferation, migration, and invasion of BCa cells (32). LncRNA OXCT1-AS1 (OXCT1 antisense RNA 1) is located on chromosome 5p13.1 and was also significantly up-regulated in BCa cell lines with LNM and was found to be inhibiting miR-455-5p in order to up-regulate the expression of JAK1, thus promoting the invasion of BCa (33).

Some lncRNAs regulate VEGF-C to promote the progression of BCa. BLACAT2 (bladder cancer-associated transcript 2) was significantly overexpressed in BCa patients with LNM. It combines with the VEGF-C promoter by forming triplexes to up-regulate VEGF-C expression, thereby promoting lymphangiogenesis and lymphatic metastasis. BLACAT2 directly interacted with WDR5 (the core component of the histone H3K4 methyltransferase complex) to epigenetically induce lymphangiogenesis and invasion (34). LNMAT1 (lymph node metastasis-associated Transcript 1) was significantly up-regulated in BCa with LNM. LNMAT1 recruited hnRNPL to the CCL2 promoter to activate CCL2 expression, resulting in increased H3K4 trimethylation, thereby ensuring hnRNPL binding and enhancing transcription. In addition, LNMAT1-induced CCL2 regulated the tumor microenvironment in BCa tissues through tumor-associated macrophages (TAMs) infiltration and VEGF-C upregulation, which ultimately led to lymphangiogenesis and lymphatic metastasis (35).

Several lncRNAs promote the progression of BCa by regulating ZEB1 and ZEB2. LncRNA SNHG16 (small nucleolar RNA host gene 16) is encoded by a 7571‐bp region at chromosome 17q25.1. Previous studies have found that SNHG16 was highly expressed in BCa tissues and was positively correlated with LNM (37). SNHG16 can regulate the proliferation, apoptosis, EMT, invasion, and migration of BCa by directly acting on the miR-17-5p/metalloproteinase 3 (TIMP3) axis, miR-200a-3p/ZEB1/ZEB2 axis, and miR-98/STAT3/Wnt/β-catenin pathway axis (36–38). LncRNA ZFAS1 (zinc finger antisense 1), located on the antisense strand of the ZNFX1 promoter region, is transcript antisense to the 5′- end of the gene zinc finger NFX1-type containing 1 (ZNFX1). Yang et al. (39) found that the expression level of ZFAS1 in BCa was increased and positively correlated with LNM. ZFAS1 can promote the proliferation, migration and invasion of BCa by down-regulating the expression of KLF2 and NKD2, and at the same time, up-regulating the expression of ZEB1 and ZEB2. It also promotes tumorigenesis of BCa through sponging miR-329 (99).

Also, some lncRNAs regulate EMT to promote BCa progression. LncRNA DLX6-AS1 (distal-less homeobox 6 antisense 1) is regulatory of members in the DLX gene family, which is localized on chromosome 7q21.3. DLX6-AS1 was up-regulated in BCa, which was related to LNM. Overexpression of DLX6-AS1 promoted the proliferation, invasion, and migration of BCa cells by regulating EMT and Wnt/β-catenin signaling pathway activity (41). DLX6-AS1-mediated miR-223 silencing can promote the growth and invasion of BCa through the up-regulation of HSP90B1 (40). LINC01296 is a novel intergenic lncRNA located at 14q11.2. The expression of LINC01296 was positively correlated with lymph node-positive BCa, and its up-regulated expression can promote BCa cells metastasis by activating the EMT pathway (42).

Another lncRNA, DANCR (differentiation antagonizing non-protein coding RNA), is located on chromosome 4q12.5, which is mainly distributed in the cytoplasm. Chen et al. (43) found that DANCR was significantly up-regulated in BCa tissues and positively correlated with LNM. DANCR promoted the LNM and BCa cells’ proliferation *via* DANCR guided LRPPRC (leucine-rich pentatricopeptide repeat containing) to stabilize its mRNA, then to activate IL-11-STAT3 signaling and increase CCND1 and PLAU expression. Zhan et al. (44) found that DANCR positively regulated the expression of MSI2 (musashi RNA binding protein 2) through sponging miR-149 to promote the malignant phenotype of BCa cells. Zhao et al. (100) found that the expression level of SPRY4-IT1 in BCa tissue was also higher than that of adjacent non-tumor tissues and was associated with LNM. SPRY4-IT1 is derived from the intron region of the SPRY4 gene and may contain several long hairpin secondary structures, which are located in 5q31.3. SPRY4-IT1 can promote proliferation and metastasis of BCa cells by sponging miR-101-3p to actively regulate the expression of EZH2 (45). Wu et al. (46) found that lncRNA NNT-AS1 was up-regulated in BCa, which was significantly associated with LNM. NNT-AS1 (nicotinamide nucleotide transhydrogenase antisense RNA 1) is located on chromosome 5p12 with 3 exons. NNT-AS1 promoted the proliferation, migration, and invasion of BCa cells by acting as a competing endogenous RNA for miR-496 to enhance the expression level of HMGB1. NNT-AS1 also targeted the miR-1301-3p/PODXL axis and activated the Wnt pathway, thereby enhancing BCa cells’ growth (47). LncRNA LNMAT2 (lymph node metastasis-associated transcript 2) was overexpressed in urinary-EXO and serum-EXO of patients with BCa, which was related to LNM. LNMAT2 was found to bind to the prospero homeobox 1 (PROX1) promoter by inducing H3K4 trimethylation, which enhanced PROX1 transcription, thus promoting lymphangiogenesis and lymph node metastasis in bladder cancer (48).

Additionally, several lncRNAs positively correlated with LNM, including: (1) HOXA-AS2, which inhibited the expression of miR-125b to promote the expression of Smad2, thus promoting the migration and invasion of BCa cells (49); (2)HNF1A-AS1, which positively regulated the expression of Bcl-2 by sponging miR-30b-5 to promote the proliferation of bladder cancer and inhibited its apoptosis (50, 101); (3) ROR1-AS1, which promoted the growth and migration of bladder cancer by regulating miR-504 (102); (4) RMRP, which promoted the proliferation, migration, and invasion of bladder cancer cells by regulating miR-206 as a sponge (103).

## The Role of circRNAs for BCa Lymph Node Metastasis

**Figure 5:**
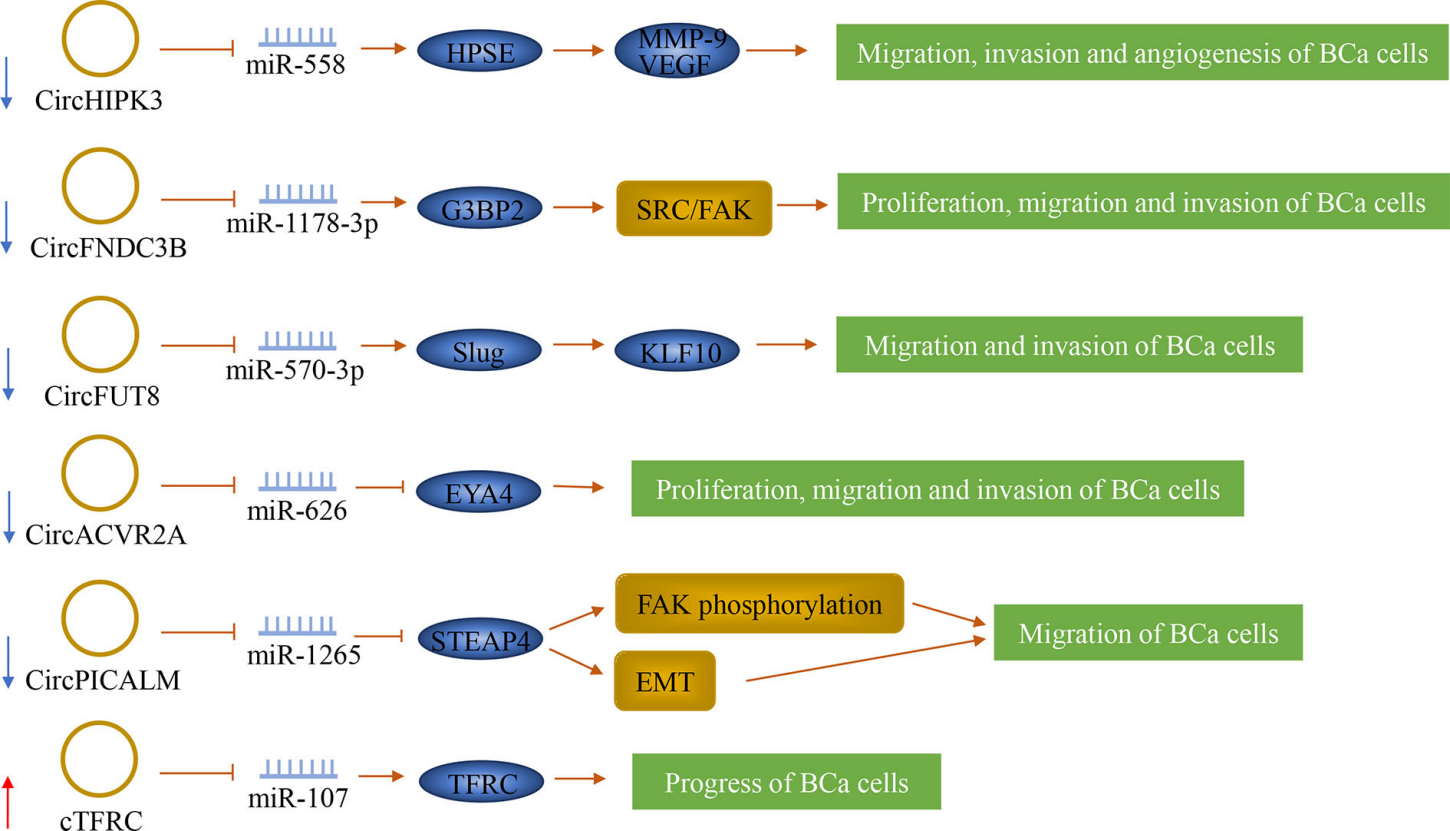
The role of circRNAs in bladder cancer lymph node metastasis. CircRNAs can play a role in bladder cancer as oncogenes and tumor suppressor genes. They can also predict lymph node metastasis.

CircRNA is a type of non-coding RNA that forms a circular structure by covalent bonds but does not have a 5’-end cap and a 3’-end poly(A) tail. It is closely related to the occurrence and development of cancer. It can act as an mRNA ‘sponge’, regulate transcription and splicing, and interact with RNA-binding proteins (104). Most circRNA negatively regulates lymph node metastasis of bladder cancer, and some molecules positively regulate this process (**Figure 5**).

CircHIPK3 (circRNA ID: hsa_circ_0000284), also known as bladder cancer-related circular RNA-2 (BCRC-2), was significantly down-regulated in BCa and was negatively correlated with LNM. It originates from the second exon of the Homeodomain-interacting protein kinase 3 (HIPK3) gene. CircHIPK3 sponged miR-558 and prevented miR-558 from being transported into the nucleus to bind the promoter of heparanase (HPSE) gene in BCa cells, thereby down-regulating the expression of HPSE and its downstream targets such as MMP-9, and VEGF, thus weakening the migration, invasion and angiogenesis of BCa cells (51). Additionally, Liu et al. (52) confirmed that circFNDC3B was significantly down-regulated in BCa tissue, and its low expression was significantly correlated with LNM. It is originated from exons 5 and 6 of the FNDC3B gene. CircFNDC3B acted as a sponge of miR-1178-3p to inhibit G3BP2 and further inhibit the downstream SRC/FAK signaling pathway, thereby inhibiting the proliferation, migration, and invasion of BCa cells.

By screening RNA sequencing data generated from human BCa tissues and matched adjacent normal bladder tissues, two novel tumor suppressors were separately identified, which are circFUT8 and circACVR2A. CircFUT8 (circBase: hsa_circ_0003028) was originated from exon 3 of the FUT8 gene. CircACVR2A was derived from exons 3, 4, and 5 of the ACVR2A gene. These two tumor suppressors were down-regulated in BCa tissues and were related to LNM (53, 54). CircFUT8 regulated the expression of Slug by sponging miR-570-3p to promote the expression of Krüpple-like-factor 10 (KLF10), thus inhibiting the metastasis and invasion of BCa cells (54). CircACVR2A can inhibit the proliferation, migration, and invasion of BCa cells by directly interacting with miR-626 and acting as a miRNA sponge to regulate EYA4 expression (53). In addition, circPICALM was found to suppress cancer progression. It is generated from exons 9-12 of PICALM. It was down-regulated in BCa tissues and associated with LNM. CircPICALM acted as a miR-1265 sponge to regulate STEAP4 and further affect FAK phosphorylation and EMT, thereby inhibiting the metastasis of BCa (55).

Serval other circRNAs were found to be possibly promoting cancer progression by inducing the malignant proliferation or migration and invasion of cancer cells. Su et al. identified a novel circular RNA called cTFRC. His study has shown that cTFRC was up-regulated in BCa tissues and was associated with LNM. The study also revealed that cTFRC might act as a sponge for miR-107 to up-regulate the expression of TFRC (transferrin receptor), further promoting the transitional phenotype of BCa cells from epithelial to mesenchymal, thereby promoting the progress of BCa. (56) Another circRNA, circPTK2, was significantly increased in BCa, and its expression level is closely related to LNM. CircPTK2 can promote the proliferation and migration of BCa cells, but its specific mechanisms are still unclear (105).

## Other Molecules as Predictive Biomarkers

In addition to the molecules described above, studies on the tumor microenvironment and genetic modification can also help predict the lymphatic metastasis of bladder cancer.

Tumors often form a microenvironment that allows inflammatory cells to proliferate and produce large amounts of mediators. D’Andrea et al. (106) found that LMR (lymphocyte-to-monocyte ratio) and NLR (neutrophil-to-lymphocyte ratio) can be used as independent factors to predict the preoperative LNM and postoperative recurrence rate of BCa patients. Zhou et al. (107) found that lymphatic vessel density (LVD) within and around the tumor increases, and lymph node metastasis of bladder cancer also increase significantly. LVD is also related to the patient’s prognosis.

m^6^A (N6-methyladenosine) refers to methylation of the N6 position of adenosine bases. m6A RNA modification is a reversible posttranscriptional modification process maintained by a multicomponent methyltransferase ‘writer’ complex (KIAA1429, METTL3, METTL14, RBM15, WTAP, and ZC3H13) and removed by demethylases ‘erasers’ (FTO and ALKBH5). The function of m^6^A in mRNA metabolism primarily depends on reader proteins, which include HNRNPC, YTHDC1, YTHDC2, YTHDF1, and YTHDF2. These regulators were differentially associated with different clinicopathological variables of BCa patients. The expression of WTAP was significantly correlated with LNM (108). Han et al. (109) found that METTL3 was significantly increased in bladder cancer and correlated with high histological grade and poor prognosis. METTL3 interacted with the microprocessor protein DGCR8 and positively modulated the pri-miR221/222 processes, resulting in the reduction of PTEN, which ultimately leads to the progression of bladder cancer.

## Predictive Models as Biomarkers for BCa LNM

The prediction model includes many aspects, such as molecules, imaging, and pathology. With the advent of models, the predictive results of bladder cancer lymphatic metastasis have become more and more reliable.

### Gene Expression Model

Smith et al. (110) developed a 20-GEM (gene expression model) for predicting pathological node status, which is evaluable on primary tumor tissue from clinically node-negative (cN0) patients. The predictive efficacy of the model is modest. Seiler et al. (111) invented a KNN51 (K-nearest neighbor classifier 51) to predict pathological lymph node metastases, but the lack of external validation limited its application. Lu et al. (112) presented a preoperative nomogram incorporating the LNM signature and a genomic mutation of MLL2. The LNM signature consists of 48 selected features. The model demonstrated good discrimination and good calibration. KNN51 included 24 non-coding features from the 51 gene signature, but the LN20 signature was based only on coding genes. Clinical factors were not incorporated into the predictive models for evaluation.

### Radiomics Nomogram

Wu et al. (113, 114) developed and validated two types of radiomics nomograms incorporating the radiomics signature and CT/MRI-reported LN status for the preoperative prediction of LNM in patients with BCa, which was a non-invasive preoperative prediction tool. It shows favorable predictive accuracy, especially for cN0 patients. Multicenter validation should be performed to acquire high-level evidence for its clinical application.

### Genomic-Clinicopathologic Nomogram

Wu et al. (115) constructed an inclusive nomogram that incorporated the five-mRNA-based classifier, image-based LN status, transurethral resection (TUR) T stage, and TUR lymphovascular invasion (LVI) to predict LNM in BCa patients. Five LN-status-related mRNAs include ADRA1D, COL10A1, DKK2, HIST2H3D, and MMP11. It shows favorable discriminatory ability and may aid in clinical decision-making, especially for cN-patients. However, it requires multicenter prospective clinical trials to provide high-level evidence for clinical application.

### Genomic-Radiomics Nomogram

Chen et al. (116) validated a genomic-radiomics nomogram incorporating CCR7 and CT to predict LNM in patients with BCa. The combined evaluation of CCR7 and CT appeared to be a more reliable marker for lymph node metastasis in BCa than the diagnosis by CT or CCR7 alone. However, these results require further confirmation by large sample and multi-center prospective studies.

## Other Factors Affecting the Prognosis of BCa

### Systemic Diseases

In recent years, studies have found that some systemic diseases were closely related to tumor occurrence and development. Metabolic syndrome (MetS) was defined as the presence of three of the following: hypertension, hyperlipidemia, diabetes, or body mass index >30. Previous studies have proved that MetS cannot predict higher pathological stages and the risks of LVI and LNM, but a single component of metabolic syndrome was related to them. Body mass index, waist circumference, and hypertension were positively correlated with the risk of higher pathological stages. And higher BMI value was related to lymphatic invasion and lymph node metastasis (117, 118). Obesity was significantly related to recurrence-free survival, cancer-specific survival, and overall mortality. Adipose tissue can produce a variety of inflammatory factors, including leptin, adiponectin, and cytokines. Leptin played an anti-tumor effect by promoting the proliferation and activation of natural killer cells (119, 120). Nonalcoholic fatty liver was positively correlated with BCa, and it was a poor prognostic factor for BCa. Patients with nonalcoholic fatty liver disease had elevated vascular endothelial growth factor, interleukin 6, TNF-α, and IGF-1. These factors may increase the risk of BCa recurrence and lead to a poor prognosis (121). Studies have shown that patients with BCa had higher insulin resistance than those without cancer but with bladder disease (122). DM was associated with elevated BCa or cancer mortality risk, especially in men (123). Metformin is the most commonly used drug for patients with t2DM. Our team’s study found that the intake of metformin was positively associated with RFS, which improved PFS and cancer-specific survival (124). Metformin targeted a YAP1-TEAD4 complex *via* AMPKα to regulate CCNE1/2 in BCa cells (125). It can suppress cyclin D1, cyclin-dependent kinase 4 (CDK4), E2F1, and mammalian target of rapamycin (mTOR) (126). The use of insulin can increase the risk of BCa progression (127). High-dose human insulin and insulin glargine similarly promoted T24 BCa cell proliferation *via* PI3K-independent activation of Akt (128).

### Environmental Toxins

Environmental toxins are closely related to cancer occurrence and development, and arsenic is the most reported in BCa. Dimethylarsinic acid (DMAV) is a methylated metabolite of arsenicals found in most mammals, and long-term exposure to DMAV can lead to BCa. Previous studies have found that recurrent BCa with high arsenic levels in tissues was more aggressive and had a higher stage and grade, and recured earlier than people with low levels of arsenic (129). Zhou et al. found that chronic arsenic exposure can upregulate HER2 in human and rat bladder epithelial cells and promote the proliferation, migration, epithelial-mesenchymal transition, and angiogenesis of cancer cells by activating the MAPK, PI3K/AKT, and STAT3 pathways (130). Moreover, sodium arsenite can reduce the human urothelial WIF1 gene expression, increase its DNA methylation level, and promote cancer cells’ migration. The WIF1 gene expression and its DNA methylation can be considered as potential biomarkers for the diagnosis of human BCa (131).

## Conclusions

For the LNM in BCa, three mechanisms are mainly involved: tumor cell proliferation, tumor cell migration and invasion, and chemosensitivity. Most biomarkers are related to the proliferation, migration, and invasion of BCa cells. Several biomarkers are involved in chemosensitivity. MiR-143, miR-101, miR-133b, MALAT1, CXCL5, and VEGF-C are related to all three of the above mechanisms. These biomarkers are more likely to be prognostic factors for BCa with LNM, but a large number of retrospective studies are still needed for further verification. Previous studies have shown that most biomarkers have a clear relationship with the prognosis of BCa patients (**Table 2**). However, the relationship between these eight biomarkers: ISYNA1, miR-539, miR-3658, OXCT1-AS1, DLX6-AS1, HOXA-AS2, circHIPK3, and circPTK2 and prognosis is still unclear; therefore, further research is needed to tap into their potential for the prognosis of BCa patients. Many biological assessment methods are economical and accurate. For example, peripheral blood can detect MMP, LMR, and NLR. Urine can detect the methylation status of GATA6 promoter, CXCL5, and MMP. Genetic testing for LNM is more sensitive and specific than traditional pathological examinations and is particularly suitable for micrometastasis diagnosis. Those test samples are easy to obtain before surgery, with strong reproducibility and high clinical feasibility. Recently, the research on SNP and m6A is also a hot spot. The relationship between them and bladder cancer with lymph node metastasis is not yet clear, and further investigation is needed, but it provides new directions for our future research. As for imaging, pathology, and molecular composition models, they are more accurate in terms of predicting lymphatic metastasis for bladder cancer, which should be studied in-depth and applied to clinical practice.

**Table 2:** The relationship between biomarkers and prognosis in bladder cancer.

Reference	Marker	Relationship with LNM	Prognosis
57	VEGF-C	Positive	DFS
64	COX-2	Positive	OS
65	PCMT1	Positive	OS
67	Sonic Hedgehog	Positive	No
69	CXCL5	Positive	OSˎPFSˎRFS
75	MMPs	Positive	OSˎRFS
76	IPO11	Positive	OS
79	SCD1	Positive	OS
–	ISYNA1	Positive	–
82	mAR-SLC39A9	Positive	OSˎDFS
88	Maspin	Negative	OSˎPFS
89	GATA6	Negative	OS
90	FOXO	Negative	OS
Chen et al. (4)	miR-101	Negative	OS
93	miR-143	Negative	OS
94	miR-133b	Negative	OSˎPFS
–	miR-539	Negative	–
18	miR-497	Negative	OS
19	miR-154	Negative	OS
21	miR-223	Negative	No
95	miR-148a	Negative	OS
–	miR-3658	Positive	–
97	LncRNA MALAT1	Positive	OS
98	LncRNA PVT1	Positive	OS
–	LncRNA OXCT1-AS1	Positive	–
34	LncRNA BLACAT2	Positive	OS
35	LncRNA LNMAT1	Positive	OSˎDFS
Peng et al. (37)	LncRNA SNHG16	Positive	OS
99	LncRNA ZFAS1	Positive	OSˎPFS
–	LncRNA DLX6-AS1	Positive	–
42	LINC01296	Positive	OS
43	LncRNA DANCR	Positive	OSˎDFS
100	LncRNA SPRY4-IT1	Positive	OS
46	LncRNA NNT-AS1	Positive	OS
	LncRNA LNMAT2	Positive	
–	LncRNA HOXA-AS2	Positive	–
Wang et al. (101)	LncRNA HNF1A-AS1	Positive	OS
Cheng et al. (102)	LncRNA ROR1-AS1	Positive	OS
103	LncRNA RMRP	Positive	OS
–	CircHIPK3	Negative	–
52	CircFNDC3B	Negative	OS
He et al. (53)	CircFUT8	Negative	OS
Dong et al. (54)	CircACVR2A	Negative	OS
55	CircPICALM	Negative	OS
56	cTFRC	Positive	OS
–	CircPTK2	Positive	–

OS, overall survival; DFS, disease free survival; PFS, progression-free survival; RFS, relapse free survival.

## Author Contributions

CZ contributed to reading the literature, preparing figures and the table, and writing the manuscript. JH, HL, HM, BO, WR, ZY, DQ, ZO, JC, and XZ assisted with writing and revised the manuscript. All authors contributed to the article and approved the submitted version.

## Funding

This work was supported by the National Natural Science Foundation of China (81873626, 81902592), Hunan Natural Science Foundation (2020JJ5884), Hunan Province Key R&D Program (2019SK2202), and Xiangya Hospital Youth Fund (2018Q09).

## Conflict of Interest

The authors declare that the research was conducted in the absence of any commercial or financial relationships that could be construed as a potential conflict of interest.
